# The effects of endogenously‐ and exogenously‐induced hyperketonemia on exercise performance and adaptation

**DOI:** 10.14814/phy2.15309

**Published:** 2022-05-25

**Authors:** David J. Dearlove, Adrian Soto Mota, David Hauton, Katherine Pinnick, Rhys Evans, Jack Miller, Roman Fischer, James S.O. Mccullagh, Leanne Hodson, Kieran Clarke, Pete J. Cox

**Affiliations:** ^1^ 6396 Department of Physiology, Anatomy and Genetics University of Oxford Headington Oxford United Kingdom; ^2^ 6396 Chemistry Research Laboratory University of Oxford Headington Oxford United Kingdom; ^3^ Oxford Centre for Diabetes, Endocrinology and Metabolism Churchill Hospital and Oxford NIHR Biomedical Research Centre University of Oxford Headington Oxford United Kingdom; ^4^ The PET Research Centre and The MR Research Centre Aarhus University Headington Oxford United Kingdom; ^5^ 6396 Clarendon Laboratory Department of Physics University of Oxford Headington Oxford United Kingdom; ^6^ 6396 Target Discovery Institute University of Oxford Headington Oxford United Kingdom

**Keywords:** ketogenic diet, ketone, ketone ester, metabolism

## Abstract

Elevating blood ketones may enhance exercise capacity and modulate adaptations to exercise training; however, these effects may depend on whether hyperketonemia is induced endogenously through dietary carbohydrate restriction, or exogenously through ketone supplementation. To determine this, we compared the effects of endogenously‐ and exogenously‐induced hyperketonemia on exercise capacity and adaptation. Trained endurance athletes undertook 6 days of laboratory based cycling (“race”) whilst following either: a carbohydrate‐rich control diet (*n* = 7; CHO); a carbohydrate‐rich diet + ketone drink four‐times daily (*n* = 7; Ex Ket); or a ketogenic diet (*n* = 7; End Ket). Exercise capacity was measured daily, and adaptations in exercise metabolism, exercise physiology and postprandial insulin sensitivity (via an oral glucose tolerance test) were measured before and after dietary interventions. Urinary β‐hydroxybutyrate increased by ⁓150‐fold and ⁓650‐fold versus CHO with Ex Ket and End Ket, respectively. Exercise capacity was increased versus pre‐intervention by ~5% on race day 1 with CHO (*p* < 0.05), by 6%–8% on days 1, 4, and 6 (all *p* < 0.05) with Ex Ket and decreased by 48%–57% on all race days (all *p* > 0.05) with End Ket. There was an ⁓3‐fold increase in fat oxidation from pre‐ to post‐intervention (*p* < 0.05) with End Ket and increased perceived exercise exertion (*p* < 0.05). No changes in exercise substrate metabolism occurred with Ex Ket, but participants had blunted postprandial insulin sensitivity (*p* < 0.05). Dietary carbohydrate restriction and ketone supplementation both induce hyperketonemia; however, these are distinct physiological conditions with contrasting effects on exercise capacity and adaptation to exercise training.

## Graphical abstract

Exercise performance and adaptive responses to an endogenously‐ and exogenously‐induced hyperketonemia are markedly different.

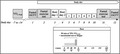

## 1 INTRODUCTION

To sustain contractile activity for extended periods, exercising skeletal muscle must rapidly resynthesize ATP through substrate‐level and oxidative phosphorylation (Hargreaves & Spriet, 2020). The relative contribution to ATP resynthesis from skeletal muscles’ primary fuels, fat, and carbohydrate (CHO), is largely dependent on the intensity of exercise undertaken (Loon et al., 2001). At moderate‐high exercise intensities (~ >70% peak oxygen uptake (VO_2 peak_)), glucose‐6‐phosphate from intramuscular glycogen catabolism represents the major fuel (Loon et al., 2001). Dependence on this relatively small energy depot is unsustainable during prolonged (~ >1 h) exercise (Pernow & Saltin, 1971).

When sufficiently elevated, the ketone bodies—β‐hydroxybutyrate (βHB) and acetoacetate (AcAc)—may provide a supplementary oxidative substrate for skeletal muscle (Balasse & Fery, 1989). Given this, it is hypothesized that inducing hyperketonemia may enhance exercise capacity by reducing muscles reliance on CHO (Cox et al., 2016; Phinney et al., 1983). However, the estimated contribution of ketone oxidation to overall energy expenditure during exercise varies widely from 0% to 18% (Balasse & Fery, 1989; Cox et al., 2016; Dearlove et al., 2021; Dearlove et al., 2021; Wahren et al., 1975), meaning the utility of this substrate is debated (Petrick et al., 2020). Ketones also have a range of metabolic signaling effects (Newman & Verdin, 2017), including altering the availability and utilization of carbohydrate and fat, which could acutely modulate exercise capacity and alter adaptative responses to exercise training (Evans et al., 2017). However, these effects may depend on whether hyperketonemia is induced endogenously or exogenously.

Blood ketone levels may be increased through severe restriction of dietary CHO (Noakes et al., 2014). With prolonged fasting, plasma βHB concentrations increase to ⁓1.5–2.5 mM within ~3 days and reach a physiological plateau of ⁓6 mM after 5–6 weeks (Robinson & Williamson, 1980). Ketogenesis may also be induced through a very‐low‐carbohydrate (ketogenic) diet, with βHB levels achieved dependent on the degree of CHO restriction (Noakes & Windt, 2016). Although the effects of a ketogenic diet on acute endurance exercise performance are equivocal, (Burke, 2021) exercise training undertaken during severe CHO restriction augments potentially favorable molecular adaptations that increase maximal fat oxidation rates (Burke, 2021). However, this adaptive response is not thought to result from hyperketonemia, but rather the metabolic milieu required to induced ketogenesis (Cox & Clarke, 2014).

Alternatively, blood βHB levels may be elevated exogenously through the consumption of ketone esters. Here, blood ketone levels rise rapidly and transiently in a dose‐dependent fashion (Dearlove et al., 2021), without the need for dietary CHO restriction. The combination of hyperketonemia with replete CHO stores may enhance acute endurance exercise capacity (Cox et al., 2016; Poffé et al., 2021), although both null (Dearlove et al., 2019; Evans & Egan, 2018; Evans et al., 2019; Poffé et al., 2020) and negative effects (Leckey et al., 2017) have also been reported. Moreover, prolonged supplementation of ketones during high‐intensity exercise training may prevent the deleterious effects of overreaching (Poffé et al., 2019), and has been hypothesized to influence the adaptive response to exercise training (Evans et al., 2017).

The comparative effects of these hyperketotic states on exercise capacity and adaptation has not been determined. Accordingly, we recruited 21 trained endurance athletes to an exercise study where exercise capacity and physiological/metabolic adaptation in response to a carbohydrate control diet, a carbohydrate‐rich diet supplemented with ketones, and a ketogenic diet was determined.

## 2 MATERIALS AND METHODS

### 2.1 Subjects

Twenty‐five participants were recruited via advertisement and direct invitation, of which seven volunteered to undertake a ketogenic diet (endogenous hyperketonemia (End Ket)). The remaining 18 subjects were randomly and single‐blindly assigned to either exogenous hyperketonemia (Ex Ket) or a CHO control (CHO). Inclusion criteria were: Aged 18–45 years; males and females (the latter must be taking the combined oral contraceptive pill); free from known disease; currently undertaking a minimum of 6 h exercise training per week; training continuously for a minimum of 3 months prior to enrolment; free from injury and illness at the time of recruitment; not currently taking medications or supplements that may interfere with lipid and glucose metabolism; and non‐smokers. Subjects were actively participating in either cycling, running, triathlon or rowing. All participants had been following a CHO‐rich diet for at least 2 months prior to enrolment. Two participants in the CHO group withdrew due to reasons unrelated to the study protocol. One participant in the CHO and Ex Ket groups withdrew due to vasovagal syncope in response to the biopsy procedures. Thus, 21 participants (*n* = 7 per group) completed the study and were included in the analyses. Participant characteristics are presented in Table 1. Ethical approval for the study was granted by the South Central—Oxford B Research Ethics Committee (17/SC/0297). All participants provided written informed consent prior to study procedures being undertaken.

**TABLE 1:** Participant characteristics

	CHO (*n* = 7)	Ex Ket (*n* = 7)	End Ket (*n* = 7)	*p*
Sex (m/f)	7/0	6/1	7/0	NA
Age (yr)	27 ± 7	25 ± 2	28 ± 7	0.7
Height (cm)	186 ± 6	189 ± 6	183 ± 13	0.4
Weight (kg)	82.2 ± 5.2	77.6 ± 8.2	79.4 ± 12.4	0.7
VO_2 Peak_ (L·min^−1^)	4.5 ± 1.1	4.3 ± 1.1	4.2 ± 0.7	0.7
VO_2 Peak_ (ml·kg bw^−1^·min^−1^)	58.0 ± 7.3	54.7 ± 11.0	53.6 ± 8.3	0.7
Power at VO_2 peak_ (W)	367 ± 44	387 ± 77	352 ± 49	0.6
HOMA‐IR	1.0 ± 0.7	1.1 ± 1.0	0.6 ± 0.2	0.2

Note

*p* values represent main group effects for each characteristic.

Abbreviations: CHO, carbohydrate; End Ket, Endogenous hyperketonemia; Ex Ket, Exogenous hyperketonemia; HOMA IR, homeostatic model assessment of insulin resistance; VO_2 peak_, peak oxygen uptake. Values are mean ± SD.

### 2.2 Dietary interventions

**FIGURE 1:**
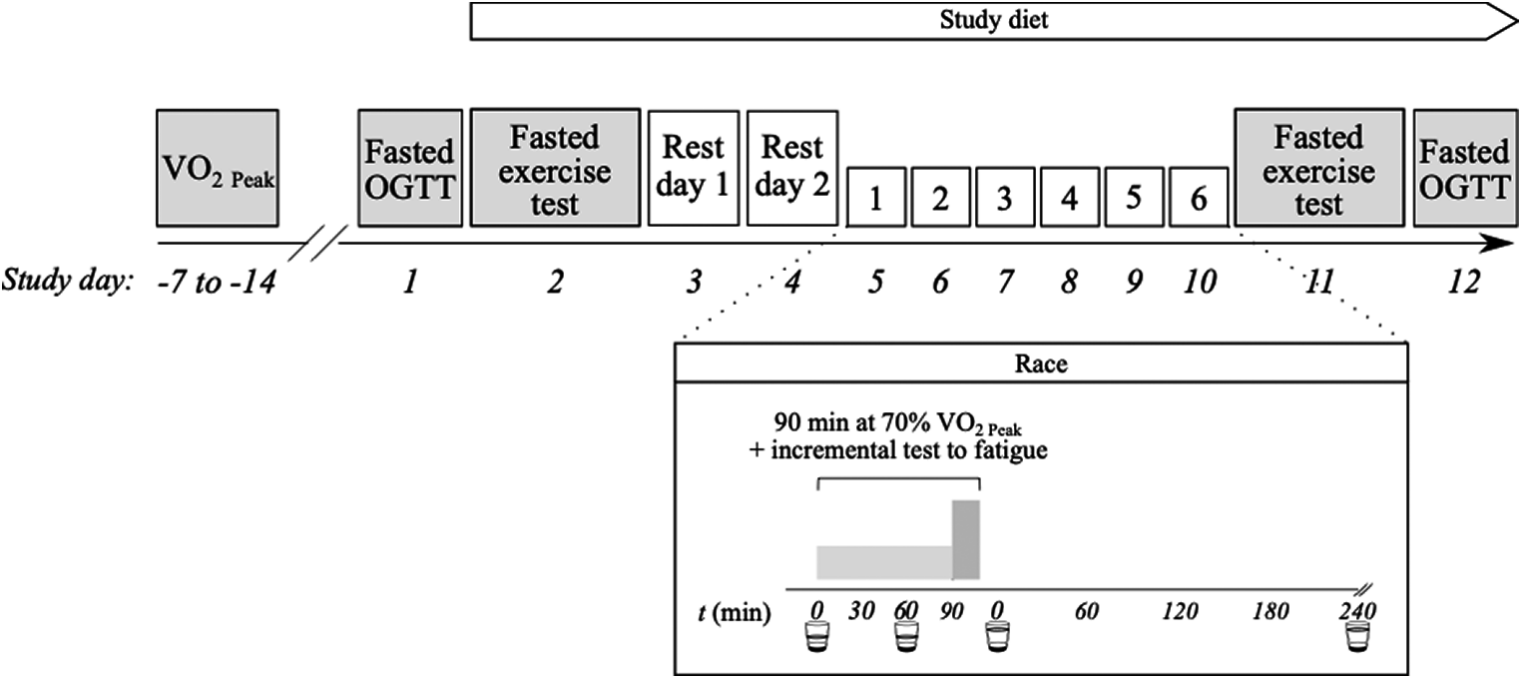
Study protocol. Three experimental diets were compared: (a) A habitual, CHO‐rich diet supplemented with a CHO‐only containing drink (CHO, *n* = 7); (b) a habitual, CHO‐rich diet supplemented with an isocaloric D‐βHB monoester and CHO containing drink (Ex Ket, *n* = 7); and (c) a ketogenic diet supplemented with an isocaloric fat containing drink (End Ket; *n* = 7). Dietary interventions began following the pre‐intervention fasted exercise test and ended at the post‐intervention OGTT. Participants completed a laboratory‐based 6‐day cycling race. Before and after the dietary intervention participants completed a fasted exercise test and OGTT. OGTT, oral glucose tolerance test; VO_2 Peak_, peak oxygen uptake.

Dietary interventions commenced immediately after the pre‐intervention fasted exercise test and ended following the post‐intervention oral glucose tolerance test (OGTT) (Figure 1). The three dietary interventions are summarized below and in Supplementary Information 1.

#### 2.2.1 CHO

As current nutritional guidelines advocate a high‐CHO diet to support endurance exercise performance and training (Thomas et al., 2016), we included a CHO control group. Participants maintained a habitual macronutrient composition diet, supplemented with drinks containing a weight‐adjusted amount of dextrose, an artificial sweetener (Neotame NutraSweet), and a bitter additive (product code: 648352, Symrise). Four study drinks were consumed daily (Figure 1).

#### 2.2.2 Ex Ket

Participants maintained a habitual macronutrient composition diet, supplemented with drinks containing a weight‐adjusted amount of D‐βHB monoester (D‐β‐hydroxybutyrate‐R 1,3‐butanediol monoester; TdeltaS), dextrose, and an artificial sweetener (Neotame NutraSweet). Based on internal piloting, we predicted this would elevate blood βHB levels for ~10 h (see Supplementary information 2).

#### 2.2.3 End Ket

To induce ketogenesis, the target dietary macronutrient composition was ~5% energy from CHO, ~80% energy from fat, and ~15% energy from protein (Noakes & Windt, 2016) (e.g., diet diary in Supplementary Information 3). Participants were given a weight‐adjusted lipid containing drink (Calogen, Nutrica HCP).

Compliance was determined through diet diaries, and in the End Ket group (non‐blinded) only, also through the measurement of pre‐breakfast capillary blood βHB concentrations using a portable monitor (Freestyle Optium Neo). βHB concentrations in 24‐h urine collections were used to determine compliance with study drink consumption in Ex Ket. Several participants reported missing one or more of their 24‐h urine collections. For this reason, urine data is reported as a ratio versus the CHO group, rather than absolute values.

### 2.3 VO_2 peak_ test

Following enrolment, subjects completed an incremental‐intensity exercise test on an electronically braked cycling ergometer (Ergoselect 100) to determine power at VO_2 peak_, as described previously (Dearlove et al., 2021). Briefly, tests began at 100 W and increased 25 W every 3 min until volitional fatigue with continuous measurements of pulmonary gases.

VO_2 peak_ was defined as the highest 20 s average for volume of oxygen consumed per min (VO_2_) during the test. Power attained at VO_2 peak_ was recorded and used to set fixed power outputs for the subsequent laboratory cycling (% of VO_2 peak_). Maximal power (W_Max_) was calculated as previously reported (Dearlove et al., 2021).

### 2.4 6‐day laboratory cycling

To assess exercise performance, participants undertook six days of laboratory exercise (‘race’; Figure 1). Participants were instructed to perform no additional exercise during this time. Participants were permitted self‐selected pre‐race meals (finished no later than 1 h before attending the laboratory). Once at the laboratory, the only energy consumed was from study drinks. The exercise consisted of 90 min at 70% VO_2 peak_ on an electronically braked cycling ergometer (Ergoselect 100). Following a 10 min break, participants undertook an incremental exercise test to fatigue. This began at 75% VO_2 peak_ and increased 5% every 5 min until volitional fatigue or inability to hold a cadence ≥60 revolutions per min (RPM).

### 2.5 Fasted exercise tests

Pre‐ and post‐intervention, participants attended the laboratory after an overnight fast for measurements of exercise metabolism and physiology (Figure 1). Body weight was measured using calibrated scales (BF508, Omron, Japan) and skin‐caliper measurements were taken at seven sites to quantify body composition (Harpenden Skinfold Caliper). A catheter was inserted into an antecubital vein of the arm and a fasted blood sample was drawn. Resting respiratory gases (via indirect calorimetry) and heart rate were recorded. A muscle biopsy was performed on the vastus lateralis using methods previously described (Dearlove et al., 2021). After a subcutaneous abdominal adipose tissue biopsy was performed (see Supplementary Information 4), participants commenced exercise. No study drinks were administered before or during exercise, so that pre‐ versus post‐intervention differences in exercise physiology/ metabolism may be attributed to the dietary intervention. The exercise protocol was identical to that employed for the exercise intervention (90 min steady state at 70% VO_2 peak_ followed by an incremental exercise test to volitional fatigue). Blood was drawn, respiratory gases and heart rates were measured, and perceived exertion was recorded at *t* = 30 min, 60 min, and 90 min during steady‐state exercise.

### 2.6 Exercise capacity

Exercise capacity was assessed as:

1. time to exhaustion at the pre‐ versus post‐intervention fasted exercise tests; and
2. time to exhaustion at the pre‐intervention fasted exercise test versus daily time to exhaustion during the race.

### 2.7 Rest days

Participants were instructed to avoid exercise on study days 3 and 4 before starting the 6‐day race (Figure 1). Study drinks were consumed on rest days.

### 2.8 Cardiorespiratory measurements

Indirect calorimetry was used to determine the volume of carbon dioxide expelled per min (VCO_2_), VO_2_, respiratory exchange ratio (RER), and expired minute volume (V_E_). Participants wore a snug, comfortable fitting face mask (Hans Rudolph) attached to a calibrated indirect calorimeter (Metalyzer 3BR2, Cortex Biophysik) for the continuous sampling of respiratory gases, displayed in real‐time using Metasoft (v7.9.1, Cortex Biophysik). Subjects wore a heart rate (HR) monitor chest strap (T31, Polar Electro) that communicated directly to the Metalyzer unit via a receiver cable attached to the bicycle ergometer.

Following the completion of measurements, cardiorespiratory data were time‐averaged (5 s) in MetaSoft and exported to a.CSV file for further analysis.

### 2.9 Calculations of carbohydrate and fat oxidation rates and gross efficiency

Energy expenditure and fat and CHO oxidation rates were calculated from VCO_2_ and VO_2_ values using the non‐protein exercise‐intensity dependent calculations of Jeukendrup & Wallis (2005). To allow direct comparison with other studies, calculations were not corrected to account for the effects of ketogenesis and ketolysis, and thus include a small systematic error (Frayn, 1983).

### 2.10 Training status

To monitor symptoms of non‐functional overreaching, participants completed the daily analysis of life demands of athletes (DALDA) questionnaire (Saw et al., 2016) between the VO_2 peak_ test and pre‐intervention fasted exercise test (‘habitual’) and prior to exercise on days 2, 4, and 6 of the race.

### 2.11 Oral glucose tolerance tests

Resting OGTTs were performed pre‐ and post‐intervention (Figure 1). Participants attended the laboratory having fasted overnight. A catheter was inserted into an antecubital vein of the arm and a baseline blood sample was taken. Participants then consumed a drink containing 75 g dextrose in 400 mL water. Blood samples were taken at *t* = 30 min, 60 min, 90 min, and 120 min while the participant rested. The Matsuda index was calculated to assess whole‐body insulin sensitivity as previously reported (Matsuda & DeFronzo, 1999).

In a sub‐sample of participants randomly selected by order of enrolment in the CHO (*n* = 3) and Ex Ket (*n* = 3) groups (see Supplementary Information 5 for sub‐sample participant characteristics and blood glucose levels during OGTTs), 500 mg of a glucose isotope tracer (D‐Glucose, [U^13^C_6_], 99%; Cambridge Isotope Laboratories) was included in the drink, providing a relative enrichment of ~0.67%. For these participants, respiratory gases were sampled using a Douglas bag immediately prior to blood draws. Participants breathed into a mouthpiece containing a one‐way valve, connected to a 50 L Douglas bag. Duplicate samples of respired gases were then collected in Exetainer vials.

### 2.12 Substrate and insulin analyses

βHB was analyzed immediately in whole blood samples using a portable analyzer (Freestyle Optium Neo, Abbott Laboratories). Plasma glucose, non‐esterified fatty acids (NEFA) and lactate were determined using a calibrated, semi‐automated benchtop analyzer (Pentra C400, Horiba Medical). Plasma insulin was assayed using an enzyme‐linked immunosorbent assay kit (Mercodia).

### 2.13 Urine collection and analysis

Participants collected urine for 24 h between the VO_2 peak_ test and pre‐intervention fasted exercise test (‘habitual’), on race day 3 and at the post‐intervention fasted exercise test. Total urine was recorded, and samples were stored at −25°C before later analysis for βHB concentration using a calibrated, semi‐automated benchtop analyzer (Pentra C400, Horiba Medical).

### 2.14 Determination of breath enrichment

^13^CO_2_ enrichment in breath was calculated as follows:

$$\begin{array}{c} \begin{array}{c} {\text{Breath enrichment = TTR CO}}_{2\exp\text{ired}}‐{\text{TTR CO}}_{2\text{background}}\\ {\text{VCO}}_{2}\left(\text{mmol}\cdot {\text{min}}^{‐1}\right)={\;\text{VCO}}_{2}\left(L\cdot {\text{min}}^{‐1}\right)\times 1000/22.4\\ {\text{Expired}}^{13}{\text{CO}}_{2}\left(\text{mmol}\cdot {\text{min}}^{‐1}\right)={\text{VCO}}_{2}\left(\text{mmol}\cdot {\text{min}}^{‐1}\right)\\ \;\times \text{breath enrichment} \end{array} \end{array}$$

where TTR background represents the enrichment of fasted (pre‐drink) samples. Expired CO_2_ enrichment was established through gas chromatography‐combustion‐isotope ratio mass spectrometry, using previously published methods (Chong et al., 2007).

### 2.15 Intramuscular protein content

Non‐targeted proteomics was performed on muscle biopsy samples taken pre‐ and post‐intervention. Samples preparation is described in Supplementary Information 6. Samples were analyzed using liquid chromatography‐coupled mass spectrometry. Peptides were separated on an EASY‐Spray column ES803 and analyzed on a Dionex UltiMateTM 3000 UHPLC system and an Orbitrap FusionTM LumosTM platform (both Thermo Fisher Scientific). (Davis et al., 2017) Raw data were imported into Progenesis QI (Waters, UK) using default parameters. Tandem mass spectrometry data were searched using Mascot (v.2.5, Matrix Science) against the Universal Periodic Review human database. Mass tolerances were set to 10 ppm for precursor and 0.5 Da for fragment masses. Peptide‐level false discovery rate was adjusted to 1%. Peptides with a score of <20 were discarded. Data were cantered and normalized in Progenesis QI (Waters), before being extracted for further data processing in Perseus (Max‐Plank Institute of Biochemistry) (Tyanova & Cox, 2018).

### 2.16 Subcutaneous abdominal adipose tissue gene expression

We hypothesized that hyperketonemia may have altered the expression of cyclic adenosine monophosphate (cAMP) signaling pathway genes in adipose tissue. Therefore, we assessed transcript expression using quantitative polymerase chain reaction (qPCR) in pre‐ and post‐intervention subcutaneous abdominal adipose tissue samples. Total RNA was extracted using TRI‐reagent (Ambion) (Neville et al., 2011). cDNA was synthesized from 500 ng of total RNA using the High‐Capacity cDNA Reverse Transcription Kit (Applied Biosystems). qPCR was performed in triplicate using a 1/40 cDNA dilution with Taqman Assays (Thermo Fisher Scientific) and Kapa Probe Fast Mastermix (Merck and Co.). Target genes were diacylglycerol O‐acyltransferase 2 (*DGAT2*) (Hs01045913 m1), hormone sensitive lipase (*HSL*) (Hs00943410 m1), phosphodiesterase 3B (*PDE3B*) (Hs00610035 m1), perilipin 1 (*PLIN1*) (Hs00160173 m1) and solute carrier family 2, facilitated glucose transporter member 4 (*SLC2A4*) (Hs01586776 m1). Reactions were run in 384‐well plates on an ABI Prism 7900HT (Thermo Fisher Scientific). The relative expression of target transcripts was calculated using the delta Ct method (Pfaffl et al., 2002). Delta Ct values for target transcripts were normalized to the delta Ct (geometric mean) of the selected reference transcripts (Neville et al., 2011): Importin 8 (Hs00914057 m1), peptidylprolyl isomerase A (*PPIA*) (Hs99999904 m1) and TATA box (Hs00427620 m1).

### 2.17 Statistics

Data are presented as mean ± SD unless otherwise stated. For primary outcome measures, mean differences and 95% confidence intervals are stated within the text. Where data did not meet parametric test assumptions, log_10_ transformations were applied (all analyses were performed on the transformed data, but non‐transformed data are presented in Figures and Results), or appropriate non‐parametric tests were employed. A one‐way ANOVA (± repeated measures) was performed in Graphpad Prism (version 9.3.1; Graphpad Software; USA) where a single independent variable containing multiple levels was present. A two‐way ANOVA (± repeated measures) or mixed effects model was performed in Graphpad Prism when multiple independent variables were present. In all cases, appropriate post‐hoc comparisons were only undertaken following confirmation of main or interaction effects. Two‐way ANOVAs on some pulmonary gas and blood substrate variables identified differences between conditions at the pre‐intervention fasted exercise test. To account for this, these data were analyzed using mixed effects models in R (RStudio version 1.3.1093; R‐project, Austria) using the ‘lme4’ package (Bates et al., 2015) with participants included as a random effect. Values for substrate metabolism, exercise economy and exertion, and blood substrate concentrations included 30 min and 60 min values only; 90 min values were excluded as only one participant in the End Ket group reached this time point at the post‐intervention fasted exercise test. Significance was set at *p* < 0.05.

## 3 RESULTS

### 3.1 Compliance to dietary interventions

With CHO and End Ket, total energy intake during the study was comparable to habitual diets (Table 2). With Ex Ket, participants tended (*p* = 0.05) to consume more calories during the race compared to their habitual diet. In comparison to their habitual diet, CHO intake during the study was increased by ~15% (*p* = 0.001) in the CHO group, with a reciprocal decrease in fat intake (*p* = 0.002). With Ex Ket, dietary D‐βHB accounted for ~11% of total energy intake during the study. Despite the addition of this fourth macronutrient, the relative proportions of CHO, fat, and protein consumed were comparable to participants’ habitual diets. CHO intake was decreased by ~43% (*p* < 0.001) and fat intake increased by ~48% (*p* < 0.001) versus habitual diets with End Ket.

**TABLE 2:** Self‐reported dietary compositions

	CHO	Ex Ket	End Ket
Habitual			
TEI (kcal)	3988 ± 1308	2907 ± 725	3660 ± 719
CHO (%)	53.5 ± 4.7	52.7 ± 13.9	48.5 ± 9.4
Fat (%)	34.0 ± 2.2	29.5 ± 10.4	35.7 ± 10.0
Protein (%)	12.4 ± 4.4	17.8 ± 4.2	15.7 ± 3.5
Rest (study days 3 and 4)			
TEI (kcal)	4152 ± 1220	3624 ± 985	3701 ± 451
CHO (%)	61.5 ± 4.7	51.2 ± 4.6	4.5 ± 0.9^¶^
Fat (%)	24.5 ± 5.0	24.9 ± 5.9	84.0 ± 2.5^¶^
Protein (%)	13.9 ± 0.5	12.3 ± 2.0	11.5 ± 2.7
BHB (%)	NA	12.3 ± 2.4	NA
Race			
TEI (kcal)	3967 ± 660	4318 ± 1050	4118 ± 945
CHO (%)	65.72 ± 7.69^¶^	49.5 ± 10.1	4.2 ± 1.8^¶^
Fat (%)	21.8 ± 4.7^¶^	27.6 ± 9.3	83.5 ± 4.6^¶^
Protein (%)	12.6 ± 3.0	12.4 ± 2.3	12.2 ± 3.5
BHB (%)	NA	10.3 ± 1.9	NA

Note

Significant post‐hoc comparisons (highlighted in grey): ^¶^versus habitual (within group).

Abbreviations: CHO, carbohydrate; End Ket, Endogenous hyperketonemia; Ex Ket, Exogenous hyperketonemia; TEI, total energy intake. Values are mean ± SD.

**FIGURE 2:**
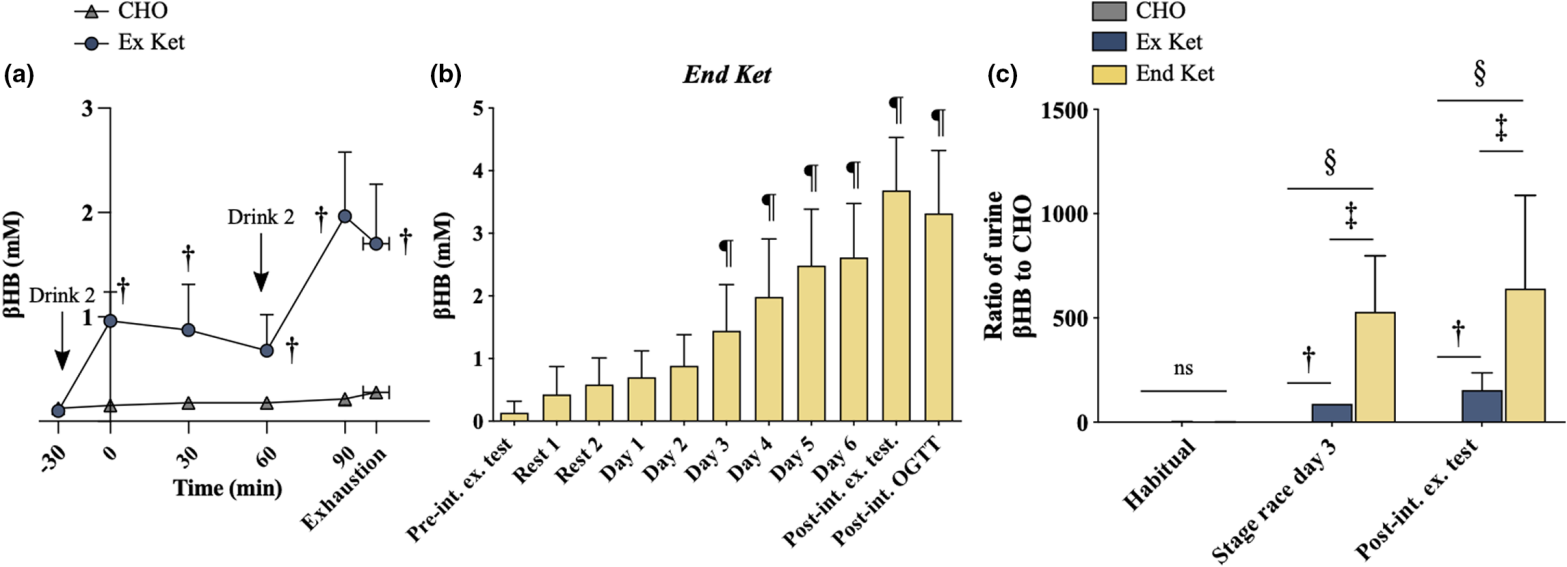
Dietary compliance. (a) Capillary βHB concentrations were measured during day 1 of the race in Ex Ket and CHO. ^†^significant difference versus CHO. (b) Overnight fasted capillary βHB in End Ket. ^¶^significant difference versus pre‐intervention fasted exercise test. (c) 24‐h urinary βHB. Significant difference between: ^†^Ex Ket and CHO; ^‡^Ex Ket and End Ket; ^§^End Ket and CHO. CHO, carbohydrate; End Ket, endogenous hyperketonemia; Ex Ket, exogenous hyperketonemia; Pre‐int. ex. test, pre‐intervention fasted exercise test; Post‐int. ex. test, post‐intervention fasted exercise test; Post‐int. OGTT, post‐intervention oral glucose tolerance test. Data presented in graphs are mean ± SD.

To confirm that the D‐βHB monoester elevated blood βHB levels, capillary βHB was measured on day 1 of the 6‐day race. Blood βHB concentrations were greater with Ex Ket versus CHO at all‐time points following consumption of the study drinks (Figure 2(a)). Abstinence from dietary CHO was sufficient to stimulate ketogenesis in End Ket, with overnight fasted capillary βHB concentrations being elevated from day 3 onwards and peaking at 3.7 ± 0.8 mM at the post‐intervention fasted exercise test (Figure 2(b)). In all groups, 24‐h urinary βHB in “habitual” (pre‐study) samples were negligible (Figure 2(c)). Urinary βHB levels were elevated in End Ket versus Ex Ket and Ex Ket versus CHO during the dietary intervention.

### 3.2 Body weight and composition

Overnight fasted body weight was the same in all groups at baseline and was unaffected by CHO and Ex Ket dietary interventions (data not shown). With End Ket, fasting body weight decreased by 3.1 ± 1.0 kg (*p* < 0.001) from pre‐ to post‐intervention fasted exercise tests. Body composition was the same in all groups at baseline and was unaffected by all diet interventions (data not shown).

### 3.3 Exercise capacity and mood disturbance

**FIGURE 3:**
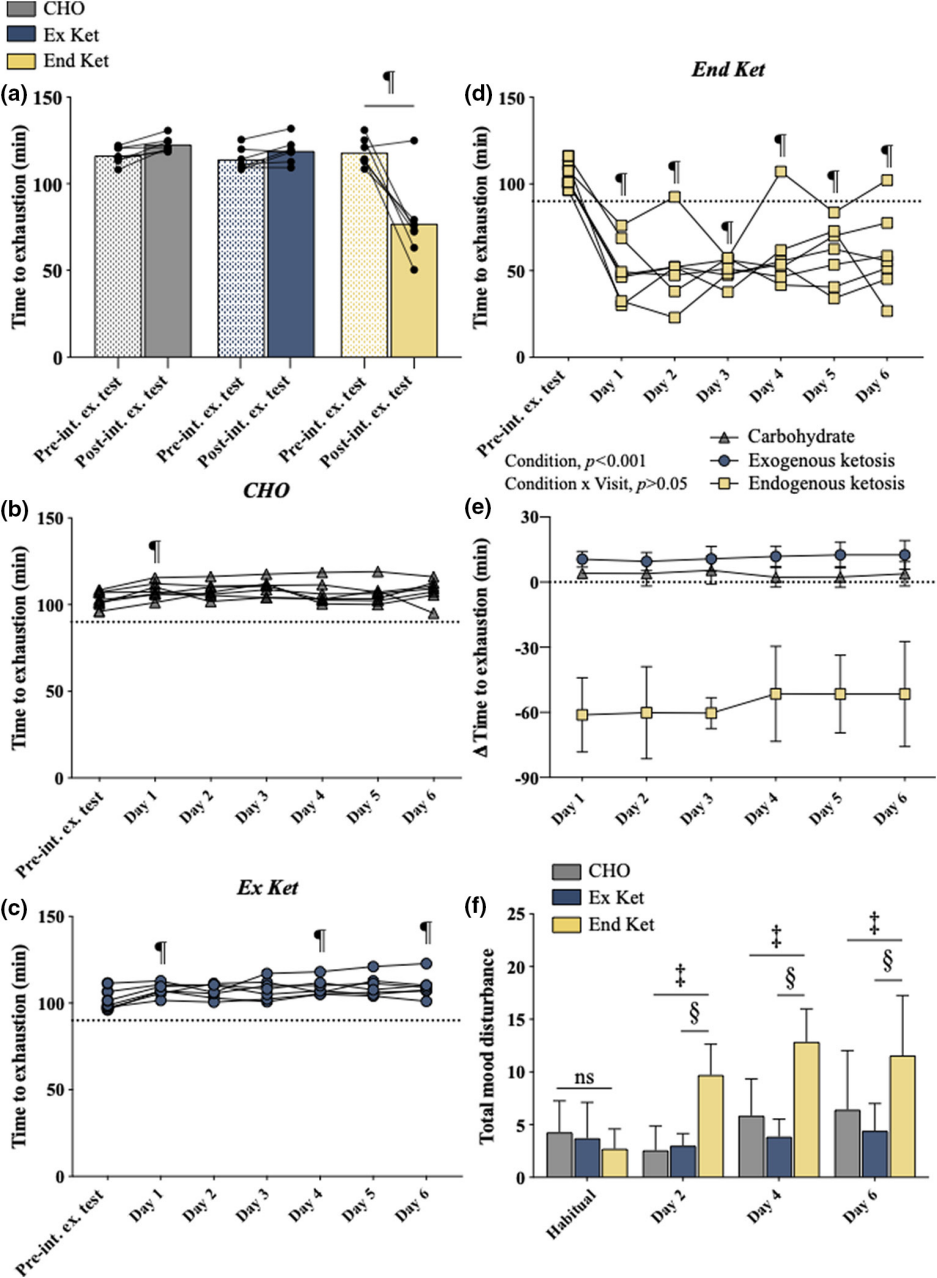
Exercise capacity. (a) Pre‐ and post‐intervention fasted exercise test time to exhaustion. ^¶^significant difference between pre‐ and post‐intervention fasted exercise tests. (b) Exercise capacity at the pre‐intervention fasted exercise test and during the race in CHO. Dotted line represents the completion of 90 min at 70% VO_2 peak_. ^¶^significant difference versus the pre‐intervention fasted exercise test. (c) Exercise capacity at the pre‐intervention fasted exercise test and during the race in Ex Ket. Dotted line represents the completion of 90 min at 70% VO_2 peak_. ^¶^significant difference versus the pre‐intervention fasted exercise test. (d) Exercise capacity at the pre‐intervention fasted exercise test and during the race in End Ket. Dotted line represents the completion of 90 min at 70% VO_2 peak_. ^¶^significant difference versus the pre‐intervention fasted exercise test. (e) Change in exercise capacity during race days versus the pre‐intervention exercise test. (f) Mood disturbance, represented by the number of “worse than normal” responses to statements in the DALDA questionnaire. Significant difference between: ^‡^Ex Ket and End Ket; ^§^End Ket and CHO. CHO, carbohydrate; DALDA, daily assessment of life demands for athletes; End Ket, endogenous hyperketonemia; Ex Ket, exogenous hyperketonemia; Pre‐int. ex. test, pre‐intervention fasted exercise test; Post‐int. ex. test, post‐intervention fasted exercise test. Data presented in graphs are mean ± SD.

Exercise capacity was comparable between groups at the pre‐intervention fasted exercise test. Pre‐ versus post‐intervention fasted exercise test capacity was unaffected with CHO (mean diff.: 5 min 56 s; CI: −7 min 12 s to 19 min 6 s) and Ex Ket (mean diff.: 4 min 35 s; CI: −8 min 32 s to 17 min 42 s) but was decreased by ~65% with End Ket (*p* < 0.001; mean diff.: −36 min 18 s; CI: −49 min 24 s to −23 min 6 s) (Figure 3(a)).

Compared to the pre‐intervention fasted exercise test, exercise capacity was greater on day 1 (mean diff. = 5 min; CI: −9 min 31 s to −29 s, *p* = 0.03) of the race with CHO (Figure 3(b)), days 1 (mean diff. = −6 min 27 s; CI: −11 min 33 s to −1 min 16 s, *p* = 0.02), 4 (mean diff. = −7 min 46 s; CI: −13 min 22 s to −1 min 44 s, *p* = 0.01), and 6 (mean diff. = −8 min 45 s; CI: −14 min 55 s to −1 min 59 sec, *p* = 0.01) with Ex Ket (Figure 3(c)) but was markedly reduced on all days with End Ket (Figure 3(d)). The change in exercise capacity during race versus the pre‐intervention exercise test was significantly different between End Ket versus Ex Ket. and End Ket versus CHO (both *p* < 0.001) (Figure 3(e)). Athletes in the End Ket group recorded more ‘worse than normal’ responses to the DALDA questionnaire versus CHO and Ex Ket on race days 2, 4, and 6 (all *p* < 0.01) (Figure 3(e)).

### 3.4 Exercise economy and exertion

VO_2_ (*p* = 0.03), V_E_ (*p* = 0.002) and energy expenditure (*p* = 0.02) were decreased with CHO during 70% VO_2 peak_ cycling at the post‐ versus pre‐intervention fasted exercise test (Table 3). HR was decreased with CHO (*p* < 0.001) and Ex Ket (*p* < 0.001) post‐ versus pre‐intervention (Table 3). RPE was increased with End Ket (*p* = 0.02) and decreased with Ex Ket (*p* = 0.02) post‐ versus pre‐intervention (Table 3).

**TABLE 3:** Exercise economy and exertion

	Carbohydrate		Exogenous ketosis		Endogenous ketosis	
	Pre‐int.	Post‐int.	Pre‐int.	Post‐int.	Pre‐int.	Post‐int.
VO_2_ (L·min^−1^)	3.77 ± 0.49	3.50 ± 0.30^¶^	3.47 ± 0.73	3.38 ± 0.74	3.30 ± 0.52	3.39 ± 0.55
V_E_ (L·min^−1^)	99.0 ± 9.4	87.1 ± 7.8^¶^	89.9 ± 16.4	90.8 ± 21.5	87.4 ± 11.7	88.7 ± 12.8
EE (J.s^−1^)	1297.2 ± 164.7	1202.5 ± 102.6^¶^	1200.7 ± 252.7	1167.4 ± 254.6	1147.2 ± 182.8	1159.7 ± 188.6
HR (BPM)	158 ± 9	147 ± 6^¶^	162 ± 8	155 ± 9^¶^	164 ± 12	165 ± 14
RPE (0–10)	5.2 ± 1.1	4.2 ± 1.0^¶^	5.6 ± 1.5	4.3 ± 1.3	5.4 ± 1.5	6.8 ± 2.0^¶^

Note

Significant post‐hoc comparisons (highlighted in grey): ^¶^versus habitual (within group).

Abbreviations: CHO, carbohydrate; EE, energy expenditure; HR, heart rate; Post‐int, post‐intervention fasted exercise test; Pre‐int, pre‐intervention fasted exercise test; RPE, rating of perceived exertion; V_E_, ventilatory exchange; VO_2_, volume of oxygen consumed.

### 3.5 Substrate metabolism

**FIGURE 4:**
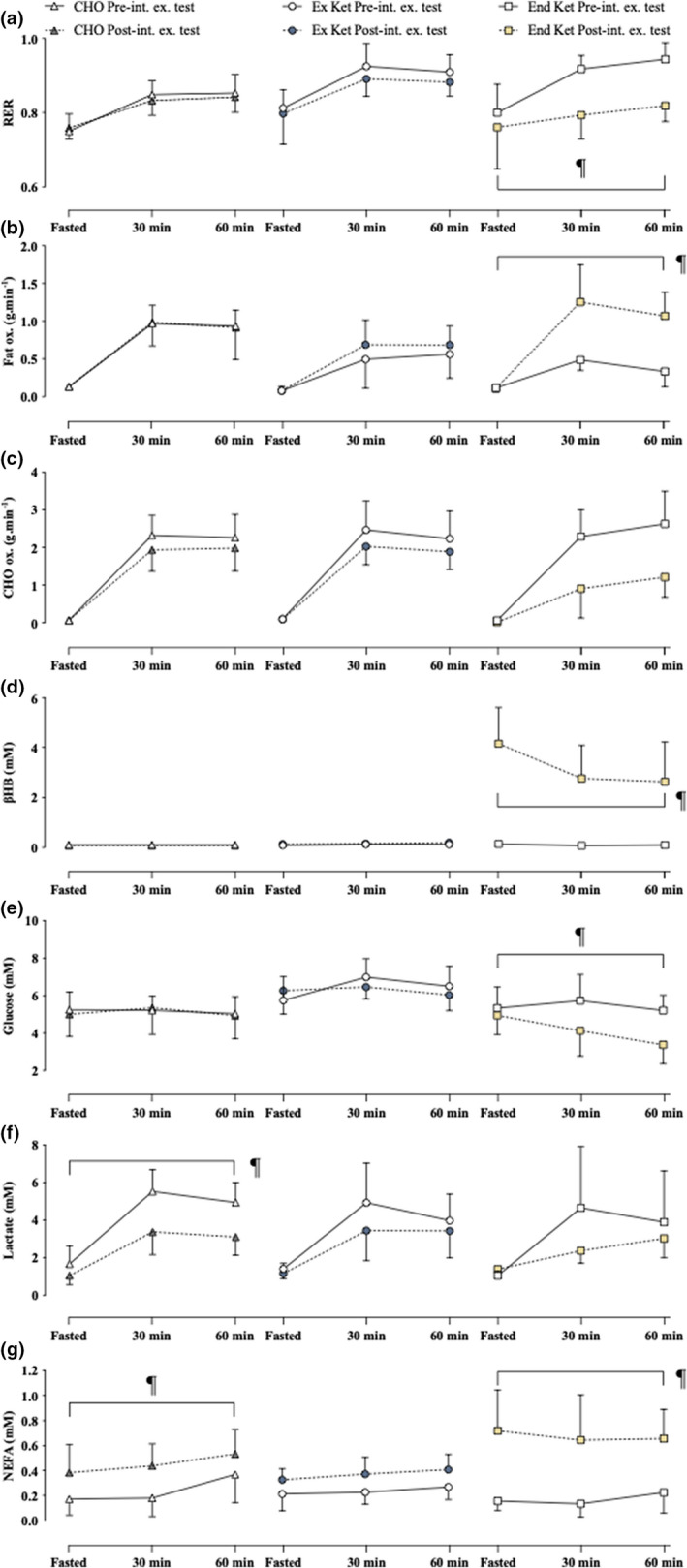
Substrate metabolism and blood substrates. (a) RER at pre‐ and post‐intervention fasted exercise tests. ^¶^significant difference versus the pre‐intervention fasted exercise test (within condition). (b) Fat oxidation rates at pre‐ and post‐intervention fasted exercise tests. ^¶^significant difference versus the pre‐intervention fasted exercise test (within condition). (c) CHO oxidation rates at pre‐ and post‐intervention fasted exercise tests. ^¶^significant difference versus the pre‐intervention fasted exercise test (within condition). (d) Plasma βHB at pre‐ and post‐intervention fasted exercise tests. ^¶^significant difference versus the pre‐intervention fasted exercise test (within condition). (e) Plasma glucose at pre‐ and post‐intervention fasted exercise tests. ^¶^significant difference versus the pre‐intervention fasted exercise test (within condition). (f) Plasma lactate at pre‐ and post‐intervention fasted exercise tests. ^¶^significant difference versus the pre‐intervention fasted exercise test (within condition). (g) Plasma NEFA at pre‐ and post‐intervention fasted exercise tests. ^¶^significant difference versus the pre‐intervention fasted exercise test (within condition). CHO, carbohydrate; CHO ox. carbohydrate oxidation rates; End Ket, endogenous hyperketonemia; Ex Ket, exogenous hyperketonemia; Fat ox., fat oxidation rates; NEFA, non‐esterified fatty acids; Pre‐int. ex. test, pre‐intervention fasted exercise test; Post‐int. ex. test, post‐intervention fasted exercise test; RER, respiratory exchange ratio. Data presented in graphs are mean ± SD.

Fat oxidation rates increased 2.8‐fold from pre‐ to post‐intervention fasted exercise tests with End Ket (*p* = 0.02), with a corresponding decrease in RER (*p* < 0.006) (Figure 4(a & b)). No changes in CHO oxidation rates were observed (Figure 4(c)).

### 3.6 Blood substrates

βHB was increased (*p* < 0.001) and glucose was decreased (*p* = 0.001) from pre‐ to post‐intervention fasted exercise tests with End Ket (Figure 4(d & e)). Blood lactate during 70% VO_2 peak_ cycling was decreased at the post‐ versus pre‐intervention fasted exercise test with CHO (*p* = 0.047) (Figure 4(f)). NEFA was decreased post‐intervention with both CHO (*p* = 0.005) and End Ket (*p* < 0.001).

### 3.7 Oral glucose tolerance test

**FIGURE 5:**
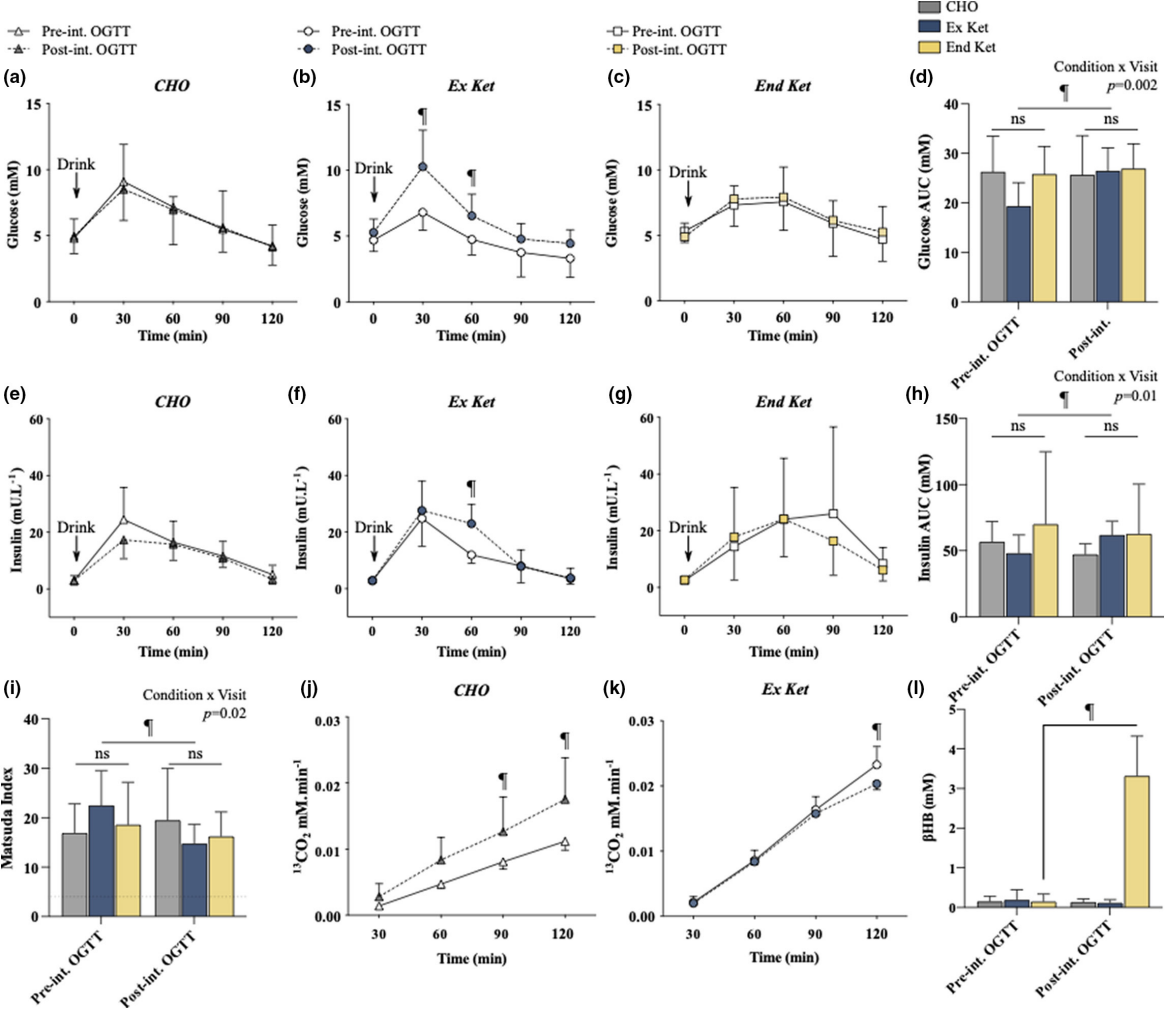
Oral glucose tolerance tests. (a) Plasma glucose concentration at the pre‐ and post‐intervention OGTT in CHO. (b) Plasma glucose concentration at the pre‐ and post‐intervention OGTT in Ex Ket. ^¶^significant difference between pre‐ and post‐intervention. (c) Plasma glucose concentration at the pre‐ and post‐intervention OGTT in End Ket. (d) Glucose area under the curve at pre‐ and post‐intervention OGTTs. (e) Plasma insulin concentration at the pre‐ and post‐intervention OGTT in CHO. (f) Plasma insulin concentration at the pre‐ and post‐intervention OGTT in Ex Ket. ^¶^significant difference between pre‐and post‐intervention. (g) Plasma insulin concentration at the pre‐ and post‐intervention OGTT in End Ket. (h) Insulin AUC at pre‐ and post‐intervention OGTTs. (i) Matsuda Index at pre‐ and post‐ intervention OGTTs. Dotted line represents the diagnostic score for insulin resistance. ^¶^significant difference between pre‐ and post‐intervention. (j) ^13^CO_2_ in respired gases at the pre‐ and post‐intervention OGTTs in CHO (*n* = 3). (k) ^13^CO_2_ in respired gases at the pre‐ and post‐intervention OGTTs in Ex Ket (*n* = 3). ^¶^significant difference between pre‐ and post‐intervention. (l) Overnight fasted (t = 0 min) blood βHB at pre‐ and post‐intervention OGTTs. AUC, area under the curve; CHO, carbohydrate; End Ket, endogenous hyperketonemia; Ex Ket, exogenous hyperketonemia; Post‐int. OGTT, post‐intervention oral glucose tolerance test; Pre‐int. OGTT, pre‐intervention oral glucose tolerance test. Data presented in graphs are mean ± SD.

In response to a 75 g oral glucose load, plasma glucose, and insulin concentrations were unaffected with CHO (Figure 5(a, d, e, and h)) and End Ket (Figure 5(c, d, g, and h)), but were markedly increased from pre‐ to post‐intervention OGTTs with Ex Ket (Figure 5(b, d, f, and h)). In line with these changes, the Matsuda Index increased (*p* = 0.02) from pre‐ to post‐intervention OGTTs with Ex Ket only.

**FIGURE 6:**
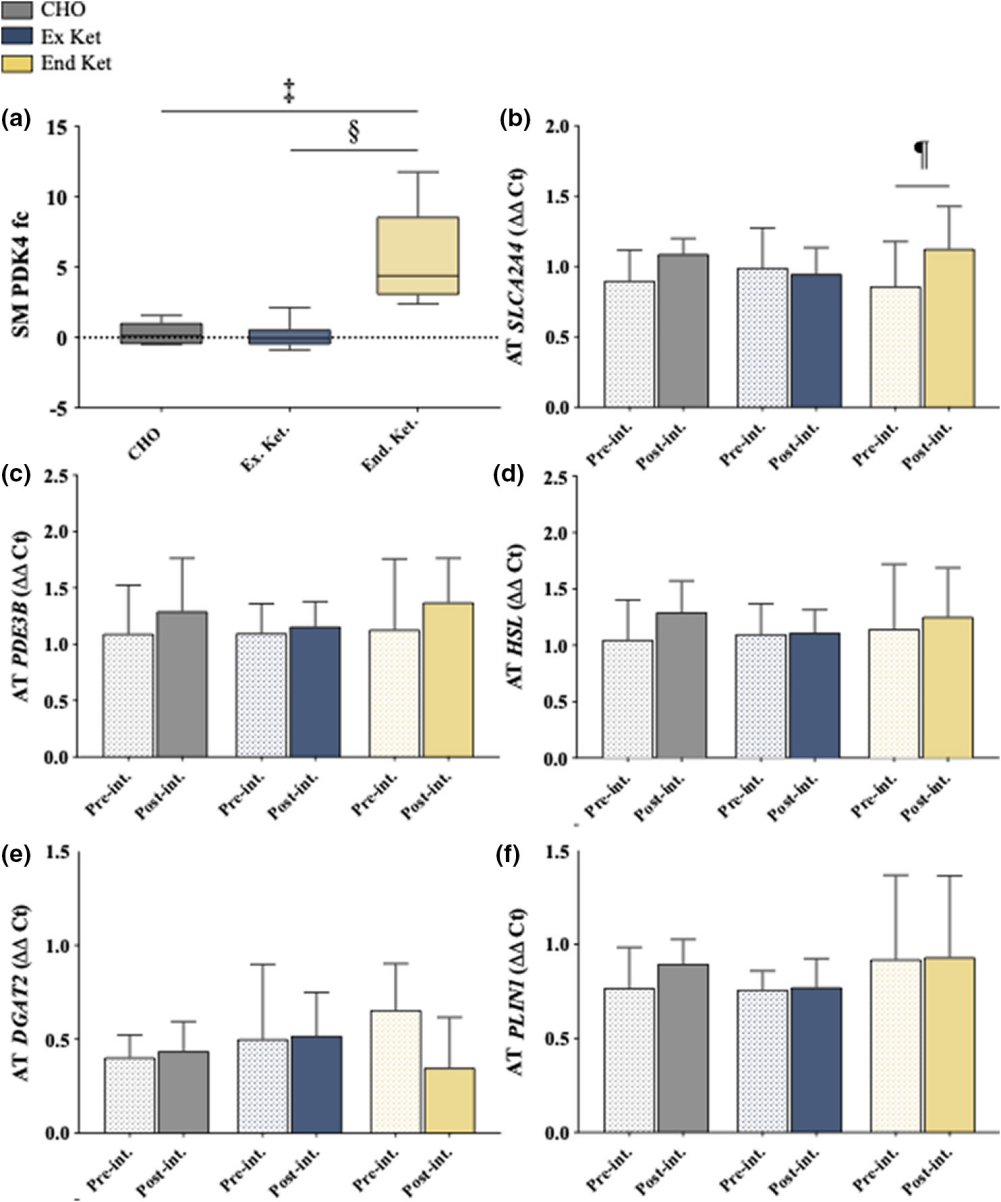
Skeletal muscle protein and abdominal subcutaneous adipose tissue gene transcripts. (a) Skeletal muscle pyruvate dehydrogenase kinase 4 (PDK4) fold changes from pre‐ to post‐intervention. Significant difference between: ^‡^Ex and End Ket; ^§^End Ket and CHO. (b) Abdominal subcutaneous adipose tissue *SLC2A4* gene expression pre‐ and post‐intervention. ^¶^significant difference between pre‐ and post‐intervention. (c) Abdominal subcutaneous adipose tissue *PDE3B* gene expression pre‐ and post‐intervention. (d) Abdominal subcutaneous adipose tissue *HSL* gene expression pre‐ and post‐intervention. (e) Abdominal subcutaneous adipose tissue *DGAT2* gene expression pre‐ and post‐intervention. (f) Abdominal subcutaneous adipose tissue *PLIN1* gene expression pre‐ and post‐intervention. AT, adipose tissue; CHO, carbohydrate; End Ket, endogenous hyperketonemia; Ex Ket, exogenous hyperketonemia; fc, fold change; Post‐int., post‐intervention; Pre‐int., pre‐ intervention; SM, skeletal muscle. Data presented in graphs are mean ± SD.

^13^CO_2_ production measured in a sub‐sample of participants (*n* = 3 Ex Ket, *n* = 3 CHO) was significantly greater at the post‐ versus pre‐intervention OGTT with CHO (Figure 6J), indicating increased exogenous glucose oxidation. Conversely, ^13^CO_2_ production at 120 min was lower at the post‐ versus pre‐intervention OGTT with Ex Ket (Figure 5(h)), indicating modestly decreased exogenous glucose oxidation. Blood glucose levels during the OGTT for this sub‐sample of participants are presented in Supplementary Information 5 and appeared comparable to the wider sample. Fasted (t = 0 min) βHB concentrations were elevated at the post‐ versus pre‐intervention OGTT with End Ket only (both *p* < 0.001) (Figure 5(l)).

### 3.8 Muscle protein and subcutaneous adipose tissue gene expression

Skeletal muscle pyruvate dehydrogenase kinase 4 (PDK4) fold change from pre‐ to post‐intervention was significantly greater (6‐fold increase; *p* < 0.001) with End Ket versus CHO and Ex Ket (Figure 6(a)). Abdominal subcutaneous adipose tissue *SLC2A4* RNA expression was significantly increased from pre‐ to post‐intervention in End Ket (*p* = 0.03, Figure 6(b)). Target genes of lipolysis and cAMP pathways were unaffected by the dietary interventions in all groups.

## 4 DISCUSSION

Hyperketonemia may enhance physical capacity and modulate adaptive responses to exercise training (Burke, 2021; Evans et al., 2017). To determine if this depends on how hyperketonemia is induced, we compared the effects of a ketogenic diet (End Ket) and ketone supplementation (Ex Ket) on exercise capacity and physiological/ metabolic adaptation to an 11‐day dietary intervention. We found that exercise performance versus a pre‐intervention test was significantly increased on 50% of race days with Ex Ket, but markedly impaired on all race days with End Ket. End Ket caused a pronounced increase in fat oxidation during exercise, whereas Ex Ket caused a blunting of postprandial insulin sensitivity. Adaptive responses to exercise may have been diminished with both Ex Ket and End Ket versus CHO.

Consistent with previous reports (Burke, 2021), exercise capacity was significantly decreased with End Ket, both versus the pre‐intervention fasted exercise test and relative to Ex Ket and CHO conditions. Additionally, athletes that adopted a ketogenic diet experienced greater perceived exertion during exercise and overall mood disturbance, as measured by the DALDA questionnaire. The 11‐day dietary intervention was arguably too short to induce cellular adaptations required to support exercise at intensities >70% VO_2 peak_ whilst primarily utilizing fat (Noakes et al., 2014). Indeed, only skeletal muscle PDK4 content—an inhibitor of pyruvate dehydrogenase and, therefore, an important regulator of glycolytic flux (Zhang et al., 2014) ‐ was increased with End Ket. However, fat oxidation rates were ~1.2 g·min^−1^ at the post‐intervention exercise test, which is comparable to those reported in athletes who adapted to a ketogenic diet for 8 months (Webster et al., 2016). This suggests fat oxidation rates may have already reached physiological limits with End Ket. One participant improved exercise capacity at the post‐ versus pre‐intervention fasted exercise test with End Ket, whereas all other participants were markedly worse. This athlete had a lower fasted blood βHB concentration (peak 1.7 mM vs. 4.0 mM group mean), which based on self‐reported macronutrient intakes and fasting NEFA levels (the precursor for hepatic ketogenesis; 0.6 mM vs. 0.7 mM group mean), was not due to non‐compliance. Notably, fat oxidation rates at the post‐intervention fasted exercise test were lower for this individual (0.8 g·min^−1^ vs. 1.2 g·min^−1^ group mean), potentially suggesting an increased capacity for glycogen synthesis from non‐glucose precursors, thus providing substrate for glycolysis. The erogenicity of supplementing athletes with ketones with or without CHO co‐ingestion versus isocaloric CHO remains equivocal (Cox et al., 2016; Dearlove et al., 2019; Evans & Egan, 2018; Evans et al., 2019; Leckey et al., 2017; Poffé et al., 2020, 2021). The change in exercise capacity (daily time to exhaustion during ‘race’ vs. time to exhaustion at the pre‐intervention fasted exercise test) was consistently higher with Ex Ket versus CHO; however, this 3‐condition study may have been underpowered to test the statistical significance of this comparatively small difference, particularly considering the substantial variation introduced by the marked decrease in exercise capacity with End Ket. Indeed, an exploratory analysis of only Ex Ket and CHO data showed a significant increase in exercise capacity with Ex Ket.

A broad goal of endurance exercise training is to increase oxidative capacity, which necessitates enhancing the ability of the cardiovascular system to deliver oxygen and substrates to skeletal muscle (central component) and skeletal muscles’ ability to oxidize substrates (peripheral component) (Ferreira et al., 2014). In response to endurance exercise training, skeletal muscle increases its capacity to oxidize fat and has enhanced metabolic flexibility (Egan & Zierath, 2013)—that is, an increased ability to switch its proportional utilization of fat and carbohydrate depending upon their availability and/ or environmental conditions (Goodpaster & Sparks, 2017). Adaptive responses to endurance exercise in the present study diverged across dietary interventions. HR at 70% VO_2 peak_ was significantly suppressed with CHO and Ex Ket. This could represent increased cardiorespiratory system efficiency (functional adaptation) or parasympathetic overtraining (non‐functional adaptation) (Budgett, 1990). In support of the former, mood disturbance (DALDA questionnaire) and exercise capacity did not deteriorate. Alternatively, the high CHO intake may have decreased urinary sodium and chloride excretion, leading to an expansion in plasma volume (Affarah et al., 1986). In turn, this may cause a lower heart rate for a given exercise intensity (Affarah et al., 1986). VO_2_, V_E_, energy expenditure, and perceived exertion were decreased during 70% VO_2 peak_ exercise from pre‐ to post‐intervention with CHO only, which may represent an advantageous cardiorespiratory and muscular adaptation in response to the high‐CHO diet. The observed blunted adaptive response to a ketogenic diet versus CHO is consistent with previous reports in elite race walkers (Burke et al., 2017, 2020). Given the growing list of ketone signaling effects on human physiology and metabolism (Newman & Verdin, 2017), it is plausible that ketone supplementation (Ex Ket) may have blunted adaptive responses versus CHO. However, further work would be required to determine this.

Consistent with a potentially deleterious adaptive response to ketone supplementation, postprandial insulin sensitivity was blunted with Ex Ket. Notably, participants did not consume ketones immediately before/during the OGTTs and, therefore, the reduced insulin sensitivity must have been an adaptive response, rather than an acute effect of hyperketonemia. This is supported by *in vitro* work demonstrating that prolonged, but not acute, incubation of cardiomyocytes and mouse soleus muscle in βHB causes reduced glucose uptake (Tardif et al., 2001; Yamada et al., 2010). The mechanism for the reduced insulin sensitivity with Ex Ket is unclear. We hypothesized that changes in adipocyte function may have contributed. Prolonged niacin infusion induces transcriptional changes in the cAMP signaling pathway within adipocytes, which may contribute to the insulin resistance observed with prolonged niacin treatment (Oh et al., 2011). βHB has a similar signaling effect on the cAMP signaling pathway in adipocytes (Taggart et al., 2005). However, we found no changes in cAMP, lipolytic or *SLC2A4 (GLUT4)* gene expression with Ex Ket. Calorie intake during race tended to be increased compared with participants habitual intake with Ex Ket only (~700 Kcal increase with Ex Ket). This finding is consistent with previous work reporting dietary intake in free‐living individuals supplemented with the same ketone ester during endurance exercise training (Poffé et al., 2019), but is contrary to reports that acute exogenous ketone supplementation suppresses appetite (Stubbs et al., 2018). Short‐term energy surplus may impair glucose tolerance (Walhin et al., 2013) and thus, the relative increase in calorie consumption with Ex Ket could, in part, explain the decrease in insulin sensitivity. However, the effects of energy surplus on glucose tolerance are reversed when concomitant daily vigorous‐intensity exercise is performed (Walhin et al., 2013), as with the present study. Whilst the mechanism for the reduced insulin sensitivity is unclear, within the context of starvation‐induced ketosis the rational for why ketones may impair insulin‐stimulated glucose disposal is logical. This action would reduce glucose uptake in insulin‐sensitive tissues—most notably, skeletal muscle—preserving this vital metabolic substrate for the brain during environmental conditions of reduced CHO availability.

Our study is not without limitations. To ensure the study drinks were isocaloric, we supplied CHO at a rate of 1.4 g min^−1^ in the CHO group, rather than the gold‐standard 2.0 g min^−1^ (Thomas et al., 2016). Moreover, we did not supply multiple‐transportable CHOs as per gold‐standard recommendations (Thomas et al., 2016). It is possible that exercise performance with Ex Ket and CHO would have been the same, or even favor CHO, had we followed these guidelines. An important explanation for observed decreases in exercise capacity following a low‐ or very‐low‐CHO diet is the depletion of intramuscular glycogen. To prioritize muscle samples for proteomic analysis, we did not measure the effects of the dietary interventions on intramuscular glycogen content and, therefore, cannot comment on this. However, the decrease in blood glucose during exercise and rapid weight loss (~60% of total weight loss within 1 day of commencing the diet) with End Ket might suggest glycogen was depleted. Only one female completed the study, who was randomized to Ex Ket. It would be useful to determine whether sexual‐dimorphism effects performance and adaptive responses to dietary interventions. Finaly, participants volunteered for End Ket, rather than being randomized to it, which may have introduced a selection bias. However, participants reported habitually consuming a CHO‐rich diet and no baseline characteristic differences were noted versus participants in CHO and Ex Ket.

## 5 CONCLUSION

Whilst dietary carbohydrate restriction and ketone supplementation both induce hyperketonemia, these are distinct physiological conditions with contrasting effects on exercise capacity and physiological/ metabolic adaptation to exercise training.

## AUTHOR CONTRIBUTIONS

David J. Dearlove and Pete J. Cox designed the study; David J. Dearlove, Katherine Pinnick, David Hauton, Leanne Hodson, Roman Fischer, Jack Miller, and James S.O. Mccullagh analyzed data; David J. Dearlove, Adrian Soto Mota, Katherine Pinnick, David Hauton, Rhys Evans and Pete J. Cox performed the research; David J. Dearlove, Leanne Hodson, Rhys Evans and Kieran Clarke wrote the manuscript; all authors approved the manuscript.

## 6 FUNDING INFORMATION

This work was funded by TdeltaS Ltd. and through an Industrial Fellowship awarded to DJD by the The Royal Commission for the Exhibition 1851.

## CONFLICT OF INTEREST

KC is director of TdeltaS Ltd., a spin‐out company of the University of Oxford that seeks to develop and commercialize products based on the D‐β‐hydroxybutyrate monoester used in this study. DJD was employed by TdeltaS Ltd. while undertaking this work. KC and PJC are named inventors on patents related to the D‐β‐hydroxybutyrate monoester. All other authors have no conflicts to declare.

## Supporting information



Fig S1

Fig S2

Supplementary Material
