# Supplementary figures and images for: The effects of endogenously‐ and exogenously‐induced hyperketonemia on exercise performance and adaptation
Source: Physiol Rep. 2022 May 25;10(10):e15309. doi: 10.14814/phy2.15309 (PMC9133544; doi:10.14814/phy2.15309)

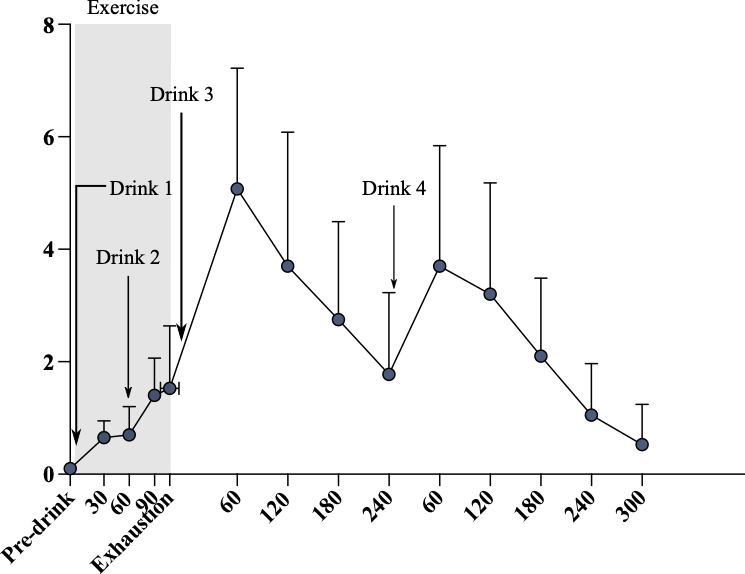

Supplement: Supplementary file 1 — Fig S1 [file PHY2-10-e15309-s001.tiff]

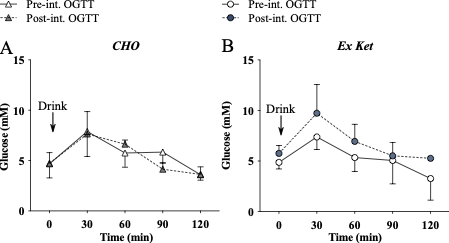

Supplement: Supplementary file 2 — Fig S2 [file PHY2-10-e15309-s003.tiff]
