# Supplementary material for: The effects of endogenously‐ and exogenously‐induced hyperketonemia on exercise performance and adaptation
Source: Physiol Rep. 2022 May 25;10(10):e15309. doi: 10.14814/phy2.15309 (PMC9133544; doi:10.14814/phy2.15309)
Supplement: Supplementary file 3 — Supplementary Material [file PHY2-10-e15309-s002.docx]

**Title:** The effects of endogenously- and exogenously-induced hyperketonemia on exercise performance and adaptation (Supplementary Information)

David J. Dearlove^1^ (david.dearlove@ocdem.ox.ac.uk), Adrian Soto Mota^1^ (adsotomota@gmail.com), David Hauton^2^ (davehauton@gmail.com), Katherine Pinnick^3^ (katherine.pinnick@ocdem.ox.ac.uk), Rhys Evans^1^ (rhys.evans@dpag.ox.ac.uk), Roman Fischer^4^ (roman.fischer@ndm.ox.ac.uk), James S.O. Mccullagh^2^ (james.mccullagh@chem.ox.ac.uk). Leanne Hodson^3^ (leanne.hodson@ocdem.ox.ac.uk), Kieran Clarke^1^ (kieran.clarke@dpag.ox.ac.uk), Pete J. Cox^1^ (petejcox456@gmail.com)

^1^Department of Physiology, Anatomy and Genetics, University of Oxford

^2^Chemistry Research Laboratory, University of Oxford

^3^Oxford Centre for Diabetes, Endocrinology and Metabolism, and Oxford NIHR Biomedical Research Centre, University of Oxford

^4^Target Discovery Institute, University of Oxford

**Running title:** Ketosis and exercise

**Corresponding author:**

David J. Dearlove

Oxford Centre for Diabetes, Endocrinology and Metabolism

Churchill Hospital

University of Oxford

Headington

Oxford

OX3 7LE

UK

Email: david.dearlove@ocdem.ox.ac.uk

Phone: +44 7739 460623

**S1. Summary of dietary interventions.**

|  | **Diet** | **Drink energy** | **Drink composition** |
| --- | --- | --- | --- |
| ***CHO*** | Habitual (carbohydrate-rich) | 3.8 kcal.kg bw^-1^ (305 ± 19 Kcal)^*^ | 100% dextrose diluted in water (400 mL total) |
| ***Ex Ket*** | Habitual (carbohydrate-rich) | 3.8 kcal.kg bw^-1^ (293 ± 31 Kcal)^*^ | 50% dextrose, 50% D-β-hydroxybutyrate monoester diluted in water (400 mL total) |
| ***End Ket*** | Ketogenic diet (~5% carbohydrate, ~80% fat, ~15% protein) | 3.8 kcal.kg bw^-1^ (294 ± 46 Kcal)^*^ | Calogen^TM^  (~4% carbohydrate, ~96% fat) |
|  |  |  |  |

Abbreviations: carbohydrate (CHO), endogenous hyperketonemia (End Ket), exogenous hyperketonemia (Ex Ket). ^*^Drinks 1 and 2 given during exercise on race days had half the calorie content.

**S2. Capillary ketone levels: Internal pilot data**

An internal pilot was performed to determine for how long blood β-hydroxybutyrate (βHB) levels would be elevated in the Ex Ket group during the race days. Four male participants (age = 47 ± 23 years, height = 186 ± 15 cm, weight = 71 ± kg) completed a single day of the race simulation, as described in the manuscript Methods (Figure 1). Blood βHB was measured using a portable monitor (Freestyle Optium Neo, Abbott Laboratories, USA).

**Figure S1. Internal pilot to determine capillary blood ketone levels during a race day in the exogenous hyperketonemia group.** Participants consumed a carbohydrate (CHO)-rich breakfast 2 h before attending the laboratory. Upon arrival, participants undertook 90 min of exercise at 70% of power at maximal oxygen uptake (VO_2 peak_). Study drinks (each was 200 mL and contained 196.5 mg·kg bw^-1^ D-β-hydroxybutyrate-R 1,3-butandiol monoester (TdeltaS, UK) and an isocaloric amount of dextrose) were consumed immediately before and at 60 min during steady-state exercise. Upon completion of steady-state exercise, participants undertook incremental-intensity exercise to fatigue (commencing at 75% VO_2 peak_ and increasing 5% VO_2 peak_ every 5 min). A third study drink (400 ml, 393 mg·kg bw^-1^ D-β-hydroxybutyrate-R 1,3-butandiol monoester and an isocaloric amount of dextrose) was consumed within 5 min post cessation of the incremental-intensity exercise. Participants left the laboratory with instructions to perform no further exercise and to follow their habitual, CHO-rich diet. A fourth and final drink (identical composition to drink 3) was consumed 4 h post-ingestion of drink 3. Capillary βHB levels were measured: immediately before and *t* = 30 min, 60 min and 90 min post drink 1; immediately upon cessation of the incremental-intensity exercise; immediately before and *t* = 60 min, 120 min, 180 min and 240 min post drink 3; and *t* = 60 min, 120 min, 180 min and 240 min post drink 3.

**S3. Example daily diet diary for a participant in the Endogenous hyperketonemia group**

| **Item** | **Amount/ volume** | **Fat** | **CHO** | **Protein** | **Kcals** |
| --- | --- | --- | --- | --- | --- |
|  |  | (kcal) | (kcal) | (kcal) | (total) |
| Coffee with cream | 30 ml | 137 | 2 | 2 | 141 |
|  | 1 cup | 0 | 11 | 0 | 11 |
| Hard-boiled egg | 1 | 37 | 0 | 30 | 67 |
| Sainsbury's coconut milk | 193 ml | 316 | 22 | 7 | 345 |
| Tesco chia seeds | 13g | 41 | 2 | 10 | 53 |
| Desiccated coconut | 10g | 58 | 3 | 3 | 64 |
| Coffee with cream | 30 ml | 137 | 2 | 2 | 141 |
|  | 1 cup | 0 | 11 | 0 | 11 |
| Koko unsweetened coconut milk | 500 ml | 72 | 4 | 4 | 80 |
| Macadamia nuts | 100g | 682 | 21 | 32 | 735 |
| Coffee with cream | 30 ml | 137 | 2 | 2 | 141 |
|  | 1 cup | 0 | 11 | 0 | 11 |
| Asparagus | 77 g | 5 | 6 | 9 | 20 |
| Smoked mackerel filets | 80 g | 184 | 2 | 72 | 258 |
| Rocket | 60 g | 3 | 9 | 5 | 17 |
| Closed cup mushrooms | 102 g | 2 | 1 | 4 | 7 |
| Halloumi | 76 g | 168 | 2 | 67 | 237 |
| Hard-boiled egg | 2 | 74 | 0 | 60 | 134 |
| Tea with 2 tbsl extra thick double cream | 30 ml | 137 | 2 | 2 | 141 |
| Mayonaise | 30 g | 189 | 19 | 1 | 209 |
| Lurpak butter | 13 g | 96 | 0 | 0 | 96 |
| Avocado | 1 med | 189 | 48 | 12 | 249 |
| Tea with 2 tbsl extra thick double cream | 30 ml | 137 | 2 | 2 | 141 |
|  |  | **2801** | **182** | **326** | **3309** |

**S4. Subcutaneous adipose tissue biopsy procedure**

A site lateral and inferior to the umbilicus was cleaned with 0.5% chlorhexidine spray (Hydrex, Ecolab, UK) and prepared. Approximately 1 mL of local anaesthetic (1% Lidocaine hydrochloride without adrenaline, Hameln Pharmaceuticals, UK) was injected, ensuring good dermal infiltration. A small incision was made through the dermis using a scalpel blade (No. 11, Swann Morton, UK). A 14-gauge needle (14 G x 8 cm, B. Braun Medical, UK) attached to a 10 mL syringe (BD Plastipak 3-Part Luer Slip Syringe, BD, USA) containing 3 mL of saline (Injection BP 0.9% w/v, Kent Pharmaceuticals, UK) was inserted through the incision. The syringe plunger was withdrawn to create suction, and a ‘sawing’ motion applied to disrupt and collect adipocytes held within the fascia. Once ~100 mg of adipose tissue was attained, each sample was washed with saline (Injection BP 0.9% w/v, Kent Pharmaceuticals, UK), transferred to an Eppendorf tube and frozen in liquid nitrogen (within ~2 min of sampling). Samples were stored at -80 ºC until further processing.

**S5. Participant characteristics for the sub-sample of participants in which glucose oxidation rates were measured during an OGTT**

|  | **CHO (n=3)** | **Ex Ket (n=3)** |
| --- | --- | --- |
| *Sex (m/f)* | 3/0 | 3/0 |
| *Age (yr)* | 24 ± 2 | 24 ± 2 |
| *Height (cm)* | 186 ± 2 | 186 ± 2 |
| *Weight (kg)* | 78.6 ± 4.2 | 78.6 ± 4.2 |
| *VO_2 Peak_ (L·min^-1^)* | 4.7 ± 0.9 | 4.7 ± 0.9 |
| *VO_2 Peak_ (ml·kg bw^-1^·min^-1^)* | 59.6 ± 7.7 | 59.6 ± 7.7 |
| *Power at VO_2 peak_ (W)* | 377 ± 17 | 377 ± 17 |
| *HOMA-IR* | 0.8 ± 0.8 | 1.2 ± 1.0 |

**Figure S2. Blood glucose curves during the OGTT for the sub-sample of participants in which glucose oxidation rates were measured during and OGTT.**

**S6. Skeletal muscle sample preparation for proteomics**

Frozen skeletal muscle samples were homogenised in lysis buffer (75 mM tris-HCl pH 6.8, 3.8% SDS, 4 M urea, 20% glycerol) using a Precellys 24 tissue homogeniser (Bertin Instruments, France). The homogenate was heated for 5 min at 90 ºC, centrifuged (13,000 g for 5 min at 4 ºC) and the supernatant removed. Protein concentrations were quantified in the supernatant using a bicinchoninic acid assay (Thermo Fisher Scientific, USA). β-mercaptoethanol (5%) was added to samples, which were then placed on a heating block for 5 min at 90 ºC. Samples were subsequently stored at -80 ºC before further extraction procedures were performed. Samples were thawed on ice and a volume containing 100 *µ*g protein was diluted in the lysis buffer, minus the SDS, for each sample (final sample volume of 50 *µ*L). A DTT reducing agent was added to samples (final concentration 5 mM; 200 mM DTT in 0.1 M tris buffer, pH 7.8), which were then incubated at room temperature for 60 min. An iodoacetamide alkylating reagent was added (final concentration 20 mM; 200 mM iodoacetamide in 0.1 M tris buffer, pH 7.8) followed by another 60 min incubation at room temperature. The total sample volume was increased to 200 *µ*L with double distilled water. Methanol (600 *µ*L) and chloroform (150 *µ*L) were added to samples before vortexing. Double distilled water (450 *µ*L) was added, and samples were again vortexed. Samples were then centrifuged (12000 g at room temperature for 1 min) and the upper aqueous phase was removed and discarded. Sample precipitation steps were repeated. Methanol (450 *µ*L) was then added to the remaining organic phase, which was subsequently vortexed and centrifuged for 2 min (12000 g at room temperature). The supernatant was removed, and the remaining protein pellets were resuspended in 50 *µ*L urea buffer (6 M urea in 0.1 M tris buffer, pH 7.8) by vortexing and sonification. Double distilled water (250 *µ*L) was added to reduce the urea concentration to <1 M. Trypsin was added in a 1:50 ratio in relation to the total protein content of samples. Samples were gently mixed and placed on a heat block set at 37 ºC for overnight digestion.

The following day, Sep-Pak C18 purification was performed. Sep-Pak columns were attached to a vacuum manifold. Columns were flushed with 500 *µ*L of "solution B" (65% CH_3_CN, 35% MiliQ-H_2_O, 0.1% trifluoroacetic acid). "Solution A" (1 mL; 98% MiliQ-H_2_O, 2% CH_3_CN, 0.1% trifluoroacetic acid) and the peptide digest samples were added to columns, which were then slowly flushed. Columns were washed with solution A. Finally, samples were eluted with 600 *µ*L of solution B. The collected eluate was then dried down in a speed vac and resuspended in 20 *µ*L of solution A.
